# Quantitative CT lung volumetry and densitometry in pediatric pectus excavatum

**DOI:** 10.1371/journal.pone.0299589

**Published:** 2024-07-23

**Authors:** Yeong Ran Song, Soo Ah Im

**Affiliations:** Department of Radiology, College of Medicine, Seoul St. Mary’s Hospital, The Catholic University of Korea, Seoul, Republic of Korea; Sapienza University of Rome: Universita degli Studi di Roma La Sapienza, ITALY

## Abstract

The purpose of this study was to evaluate the quantitative computed tomography (CT) volumetry and densitometry and in pediatric patients with pectus excavatum (PE). We measured pectus index (PI) and separated inspiratory and expiratory lung volumes and densities. We obtained the total lung volume (TLV) and mean lung density (MLD) during inspiration and expiration, and the ratio of end expiratory to inspiratory volume (E/I volume) and MLD (E/I density) were calculated. The difference between inspiratory and end expiratory volume (I-E volume) and MLD (I-E density) were also calculated. A total of 199 patients, including 164 PE patients and 35 controls, were included in this study. The result shows that the PE group had lower inspiratory TLV (mean, 2670.76±1364.22 ml) than the control group (3219.57±1313.87 ml; p = 0.027). In the PE group, the inspiratory (-787.21±52.27 HU vs. -804.94±63.3 HU) and expiratory MLD (-704.51±55.41 HU vs. -675.83±64.62 HU) were significantly lower than the indices obtained from the control group (p = 0.006). In addition, significantly lower values of TLV and MLD difference and higher value of TLV and MLD ratio were found in the PE group (p <0.0001). PE patients were divided into severe vs. mild groups based on the PI cutoff value of 3.5. The inspiratory MLD and TLV ratio in the severe PE group were lower than those in the mild PE group, respectively (p <0.05). In conclusion, quantitative pulmonary evaluation through CT in pediatric PE patients may provide further information in assessing the functional changes in lung parenchyma as a result of chest wall deformity.

## Introduction

Pectus excavatum (PE) is the most common congenital chest wall deformity, accounting for 1/300-400 live births of incidence, with male predominance [1]. Depression of the sternum and adjacent costosternal joints is the main problem of PE. This deformity results in displacement of the lung and the heart, and may also lead to functional and physical cardiorespiratory or cosmetic problems [2, 3].

Chest computed tomography (CT) is the main assessment tool to evaluate the existence, severity and effects of the PE [4].

Recently, few studies have reported lung volume or density analysis in patients with PE using quantitative CT [5–7]. However, there still is a relative lack of comparative studies on PE, assessing lung parenchymal density in patients with many acquired pulmonary diseases such as chronic obstructive pulmonary disease and interstitial lung disease [8–11].

The purpose of this study was to evaluate the quantitative assessment of CT in pediatric PE patients by analyzing various lung volume and density indices during inspiration and expiration using the chest CT quantitative method.

## Materials and methods

### 1. Study population

We retrospectively reviewed 199 pediatric patients under 18 years of age (range, 4–18 years) who underwent 3D chest CT from October 2019 to September 2021 at the department of Pediatrics or Thoracic and Cardiovascular Surgery, Seoul St. Mary’s hospital. Inclusion criteria for PE group were 164 children who were (a) diagnosed as PE by physicians at the department of Pediatrics or Thoracic and Cardiovascular Surgery, (b) underwent 3D chest CT with pre-operative evaluation (before PE correction surgery such as the Nuss procedure), without (c) lung lesions (pneumonia, atelectasis, pulmonary fibrosis, etc) or underlying disease (prematurity, severe heart disease, etc) that affect CT quantification values in chest CT. Patients who underwent 3D chest CT without chest wall deformity were included in the control group (n = 35). Patient data with poor cooperation of inspiration and expiration (by evaluation of membranous tracheal changes) were excluded. This study was approved by the institutional review board of Seoul St. Mary’s hospital (IRB number: KC23RASI0262). The data collection date was 1 month from April 22, 2023, and the data access date for research purposes was from April 22, 2023 to May 31, 2023. In relation to the research progress, the subject’s personal information was anonymized by assigning a research code, and the subject’s anonymity was thoroughly maintained throughout the entire process.

### 2. Image acquisition

Before CT scanning, all patients received instructed multiple rounds of training to achieve full-inspiratory and end expiratory breath hold. CT scans during full inspiration and end expiration were obtained craniocaudally in the supine position throughout the entire thorax without intravenous contrast material.

All CT examinations were performed using a helical CT scanner (Somatom Force, Siemens Medical Systems, Erlangen, Germany) using a 250–350 mm field of view, a 512×512 reconstruction matrix, 80–100 kVp, effective mAs (CARE Dose 4D), and a tube rotation time of 0.25 ms. For lung parenchymal analysis, images were reconstructed using the following parameters: 1mm thickness, no interval, and Br36 kernel.

CT attenuation measurements were taken using the CT Pulmo 3D application in a 3D solution program (Syngovia; Siemens). The application also automatically calculated lung parenchymal volume and density.

### 3. Pectus index

**Fig 1:**
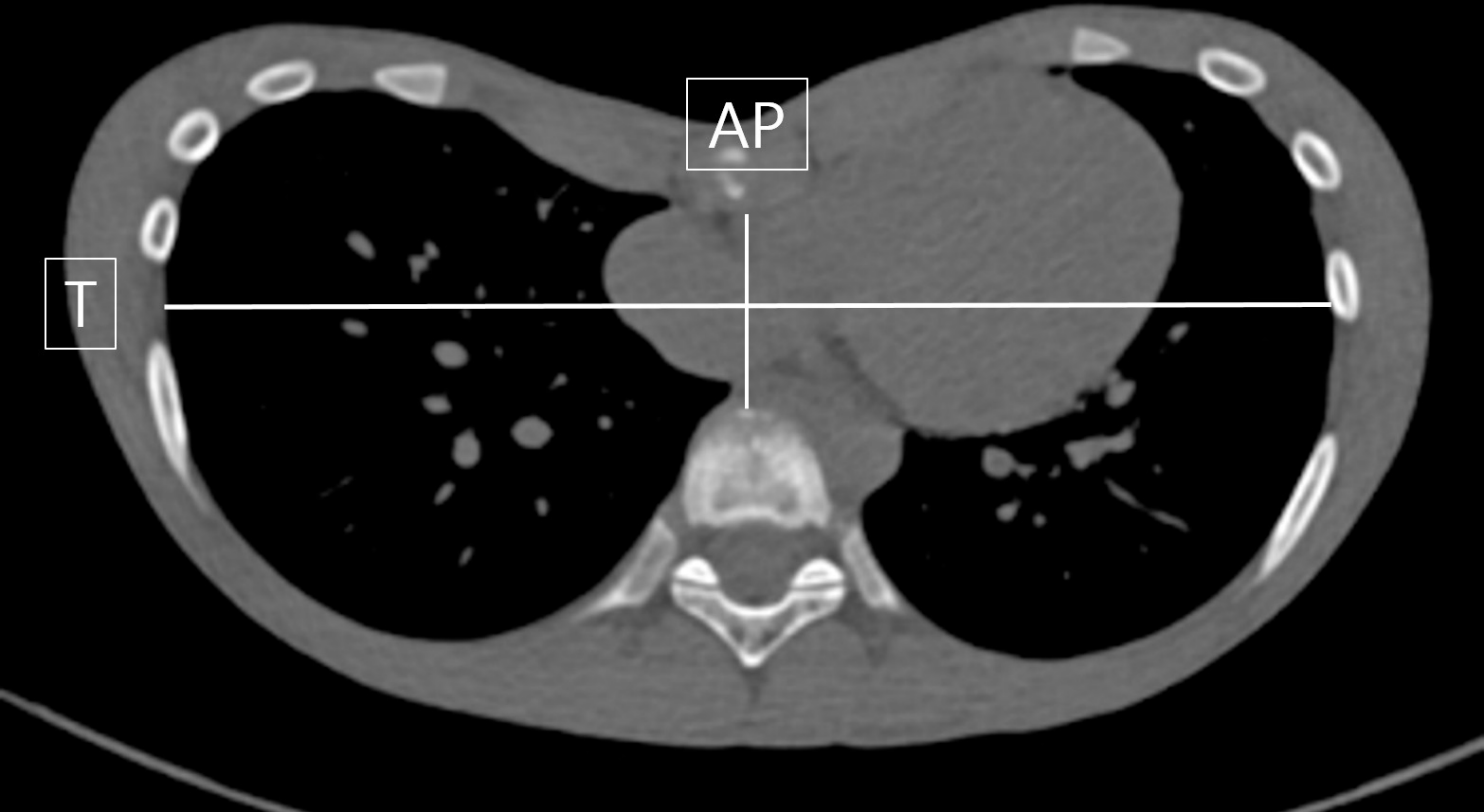
Example of an axial CT image with the measurements required for calculation of the Pectus index (T/AP).

Pectus index (PI) is the main measurement tool to assess and describe the severity of PE. PI is calculated by dividing the maximum transverse (T) diameter of the chest by the minimum anteroposterior (AP) diameter at the level of deepest depression of anterior chest wall (Fig 1). The patient’s PI was determined as the average value of the PI independently measured by two radiologists.

### 4. Lung volume and density analysis

**Fig 2:**
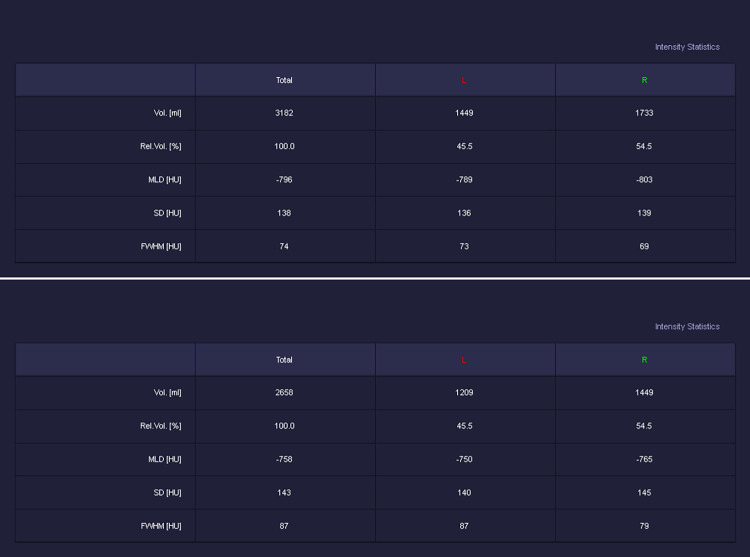
Example of volumetry and densitometry parameter measurements using the 3D chest CT of a representative subject in the A) inspiratory and B) expiratory phases.

For each side of the lungs, separated inspiratory and expiratory lung volumes and densities were measured. Based on these measurements, the total lung volume (TLV, mL) and mean lung density (MLD, HU) during inspiration and expiration were calculated (Fig 2). Furthermore, the ratio of end expiratory to inspiratory volume (E/I volume) and MLD (E/I density) and the differences between inspiratory and end expiratory volume (I-E volume) and MLD (I-E density) were calculated.

### 5. Statistical analysis

Statistical analyses were performed using the SAS system for Windows V 9.4.

Distribution of continuous variables was evaluated for normality using the Shapiro-Wilk test.

Correlation between the patients’ physical measurements and various quantitative lung volume and density indices in CT scans were compared using Spearman correlation coefficient, and linear correlation of these indices was measured using Pearson correlation coefficient.

We also compared the two groups with or without PE using Wilcoxon rank sum test because the two groups did not statistically satisfy the normal distribution using the Shapiro-Wilk test.

A p-value < 0.05 was considered to indicate statistical significance.

## Results

### 1. Patient characteristics

In this study, 164 PE patients (boys/girls, 130/34) and 35 healthy controls (boys/girls, 24/11) were included. The mean age of the PE and control groups were 10.35 years ± 4.56 (age range, 4 to 18 years) and 12.43 years ± 3 (age range, 6 to 18 years), respectively. The number of PE patients with PI < 3.5 was 43 (26.22%), while > 3.5 was 121 (73.78%) on the basis of inspiratory phase measurements. There were no patients who had any syndrome associated with PE. The detailed demographic and clinical characteristics of the patients are presented in Table 1. There were no differences in gender distribution between the PE and control groups, while the control group exhibited higher values in terms of age, height, and weight.

**Table 1:** Patient’s characteristics.

	Pectus excavatum	Control	Total	P-value
	(n = 164)	(n = 35)	(n = 199)	
**Age (years)**	10.35±4.56 (4–18)	12.43±3 (6–18)	10.71±4.4 (4–18)	0.022
**Sex (M/F)**	130/34 (79.27%)	24/11 (68.57%)	154/45 (77.39%)	0.170
**Height (cm)**	143.61±27.17 (77–188)	155.62±19 (110–193.3)	145.72±26.28 (77–193.3)	0.044
**Weight (kg)**	37.24±16.23 (8.4–76)	51.47±19.33 (20–101)	39.74±17.62 (8.4–101)	0.000
**Pectus index_ins**	4.19±1.05 (2.65–9.27)	2.24±0.16 (2.01–2.48)	4.08±1.11 (2.01–9.27)	0.000
**Pectus index_exp**	5.03±1.94 (2.57–17.19)	2.74±0.41 (2.26–3.71)	4.9±1.96 (2.26–17.19)	0.000

* mean ± standard deviation (range, min-max)

Definition of abbreviations: ins, inspiration; exp, expiration

### 2. Quantitative CT analyses

Table 2 shows the comparison results of quantitative CT analyses including lung volumetry and densitometry between the PE and the control groups.

**Table 2:** The comparison of quantitative CT parameters between the pectus excavatum and control groups.

	Pectus excavatum (n = 164)	Control	P-value
		(n = 35)	
**Vol_ins**	2670.76±1364.22 (702–6314)	3219.57±1313.87 (522–6391)	0.027
**Vol_exp**	1739.94±796.15 (463–4228)	1745.6±731.16 (434–3509)	0.792
**Density_ins**	-787.21±52.27 (632–880)	-804.94±63.3 (575–868)	0.006
**Density_exp**	-704.51±55.41 (451–825)	-675.83±64.62 (534–813)	0.006
**Vol diff (I-E)**	930.82±757.46 (15–2937)	1473.97±850.29 (88–4011)	0.000
**Density diff (I-E)**	-82.7±50.73 (3–306)	-129.11±54.4 (38–252)	< .0001
**Vol ratio (E/I)**	0.69±0.15 (0.35–0.98)	0.56±0.14 (0.31–0.83)	< .0001
**Density ratio (E/I)**	0.9±0.06 (0.6–1)	0.83±0.06 (0.71–0.96)	< .0001

* mean ± standard deviation (range, min-max)

Definition of abbreviations: Vol, volume; ins(I), inspiration(E); exp, expiration; diff, difference

Most parameters regarding inspiratory lung volume and both inspiratory and expiratory lung densities showed statistically significant difference compared to the parameters of the control group. Inspiratory TLV was significantly lower in PE (mean, 2670.76±1364.22) than those of the control group (3219.57±1313.87; p = 0.027). The inspiratory (-787.21±52.27 HU vs. -804.94±63.3 HU) and expiratory MLD (-704.51±55.41 HU vs. -675.83±64.62 HU) of the PE group were significantly lower than the indices of the control group (p = 0.006). There was no significant difference between the lung volumes during expiration.

Comparing the TLV and MLD differences and ratios between two groups during respiratory phase, significantly lower values of TLV and MLD differences and higher values of TLV and MLD ratios were found in the PE group (p-value <0.0001). These results suggest that there is little difference of TLV and MLD values between the respiratory phases in the PE group.

### 3. Correlation between quantitative CT values and clinical features

The mean age of the PE group was 10.35 years ± 4.56 (median 11 years, range 4 to 18 years) and the mean height and body weight were 143.61 cm ± 27.17 (median 150.35 cm, range 77 to 188 cm) and 37.24 kg ± 16.23 (median 35.65 kg, range 8.4 to 76 kg), respectively. There was a significant correlation between indices such as TLV and MLD and the patients’ demographic characteristics in the PE group (p<0.0001). In particular, significant relevance was found between TLV and age and between TLV and the physical measurements made in the PE group (r>0.80; ρ>0.85) (Table 3).

**Table 3:** Correlation coefficient between quantitative CT parameters and clinical features.

	Age (years)		Height (cm)		Body weight (kg)	
	r*	p-value	r*	p-value	r*	p-value
**Vol_ins**	0.90656	< .0001	0.90489	< .0001	0.88014	< .0001
**Vol_exp**	0.80997	< .0001	0.84584	< .0001	0.80966	< .0001
**Density_ins**	0.6976	< .0001	0.68094	< .0001	0.59997	< .0001
**Density_exp**	0.33971	< .0001	0.36026	< .0001	0.28086	0.0003

* Pearson’s correlation coefficient

Definition of abbreviations: Vol, volume; ins, inspiration; exp, expiration

### 4. Quantitative CT lung volume and densitometry in PE patients according to severity

To evaluate changes in lung parenchyma with respect to the severity of PE, we compared quantitative CT parameters concerning the lung volume and density between two groups which were classified by the PI value. Based on the PI value of 3.5, we divided the PE patients into severe and mild groups. Since the PI was measured during both inspiration and expiration CT, each group was compared separately.

The mean inspiration CT lung density was measured as -779.64 ± 54.90 HU and -808.49 ± 36.94 HU in the severe and mild PE groups, respectively (p<0.01). Also, there was a significant correlation between TLV ratio and PI in the PE group (0.71±0.15 vs 0.65±0.15; p<0.05) (Table 4).

**Table 4:** Lung volumetry and densitometry in pectus excavatum patients according to pectus index severity measured on inspiration CT.

Pectus index	Severe (n = 121)	Mild (n = 43)	P-value
ins phase	Mean±SD	Mean±SD	
**Vol_ins**	2589.58±1337.59	2899.19±1427.78	0.186
**Vol_exp**	1723.13±800.30	1787.23±791.77	0.471
**Density_ins**	-779.64±54.90	-808.49±36.94	0.008
**Density_exp**	-702.15±53.06	-711.16±61.76	0.209
**Vol diff (I-E)**	866.45±734.64	1111.95±799.47	0.055
**Density diff (I-E)**	-77.50±47.01	-97.33±58.1	0.053
**Vol ratio (E/I)**	0.71±0.15	0.65±0.15	0.026
**Density ratio (E/I)**	0.9±0.06	0.88±0.07	0.074

Definition of abbreviations: ins, inspiration; Vol, volume; exp, expiration; diff, difference

No significant correlations were found between quantitative CT parameters and PI severity on expiratory measurements (Table 5).

**Table 5:** Lung volumetry and densitometry in pectus excavatum patients according to pectus index severity measured on expiration CT.

Pectus index	Severe (n = 147)	Mild (n = 17)	P-value
exp phase	Mean±SD	Mean±SD	
**Vol_ins**	2713.81±1389.23	2298.47±1188.36	0.307
**Vol_exp**	1752.31±804.63	1633.00±732.12	0.714
**Density_ins**	-787.84±52.91	-781.76±47.47	0.455
**Density_exp**	-704.54±51.17	-704.24±86.02	0.562
**Vol diff (I-E)**	961.50±770.27	665.47±589.60	0.177
**Density diff (I-E)**	-83.29±48.59	-77.53±68.15	0.294
**Vol ratio (E/I)**	0.69±0.15	0.73±0.15	0.166
**Density ratio (E/I)**	0.90±0.06	0.90±0.09	0.387

Definition of abbreviations: exp, expiration; ins, inspiration; Vol, volume; diff, difference

## Discussion

In addition to cosmetic and psychological problems, PE often causes cardiorespiratory symptoms [12, 13] and requires surgical treatment depending on the severity of PE [14–16]. In evaluation of PE, chest CT is mainly used to determine the degree of severity according to the displacement of the sternum and ribs. It is also used to evaluate lung parenchymal abnormalities such as mediastinum shift, cardiac compression, atelectasis, and tracheobronchial compression. However, visual analysis have limitations in accurate lung parenchyma evaluation such as lung volume and density.

Therefore, in addition to the existing CT analysis, there have been several attempts to obtain additional information on the lung parenchyma through quantitative CT. Quantitative CT measurements of the lung volume and density are sensitive and objective methods for the evaluation of lung parenchymal and structural abnormality. So far, there have only been a few reports on pediatric PE using this quantitative CT [5, 7]. This study is meaningful in that it evaluated lung parenchymal changes in greater detail with respect to the severity of PE through quantitative CT analysis. The results show TLV and MLD were strongly correlated with patient’s age and physical measurements. Furthermore, we demonstrated that the PE group had lower mean lung density and inspiratory lung volume compared to those of healthy children with statistical significance. However, there was no difference in expiratory lung volume between the two groups, suggesting restricted pulmonary function in PE patients.

Sarioglu et al. reported that patients with PE had decreased mean lung density [5]. Singh et al. proposed a radiologic sign of the PE severity as CT finding of hyperinflation of the left lower lobe anterior basal segment [17], which was measured using local lung density in the selected area. These studies are consistent with our results on lower lung density values in the PE patients.

Since the minimally invasive technique using the Nuss bar was introduced in 1998, surgical treatment for patients with PE has been continuously evolving. Regarding the criteria for surgical treatment, there still is a lack of guidelines based on previous studies and data. Many hospitals roughly use a PI of 2.6 and 3.2 or higher for diagnosis of PE and indication for surgical treatment, respectively. However, the PI inherently has significant range of normal values depending on the patient’s gender, age, spinal level, and breathing condition. Therefore, careful judgement is required [18–21].

Currently, the treatment of patients with PE is often focused on aesthetic improvement of the sternum and ribs, with the goal of bringing the shape of the thorax closer to “normal” by increasing the sterno-vertebral distance [22]. This approach tends to neglect the evaluation of accompanying deterioration in lung function [23]. Although pulmonary function tests are used in adults for functional evaluation [12], only one study has been conducted to evaluate the association between pulmonary function tests and quantitative CT measurements in patients with PE [5].

Sarioglu et al. described a partial decrease in pixel density of the lung’s right lower lobe in patients with PE. However, lung density values were measured only at T4 and T10 levels and the CT was performed only during inspiratory phase which is insufficient for overall quantitative evaluation. For example, depression of the thoracic cavity and cardiac compression becomes worse during expiration than during inspiration in patients with PE, which is why some hospitals obtain CT images of patients with PE only at the end of expiration [18]. In the case of children, it is difficult to cooperate with pulmonary function tests (especially in children under the age of 8–9 years). Therefore, quantitative CT is thought to be a more useful tool for lung parenchymal evaluation.

In our study, the differences of TLV and MLD between the inspiratory and expiratory phases showed smaller values in the PE group while the ratios of the expiratory/inspiratory TLV and MLD showed a higher values in the PE group. Among the mild and severe groups of PE patients, the inspiratory MLD and TLV ratios were more deteriorated in the severe group. This result indicates that the variations in lung volume and density according to the phases of breathing were insufficient in the PE group compared to the control group. In other words, the lungs of the children with PE may be stiffer and more immature than those of the children without PE. Nonetheless, this hypothesis require further retrospective studies.

In conclusion, quantitative pulmonary evaluation through CT in pediatric PE patients may provide further information in assessing the functional changes in lung parenchyma as a result of chest wall deformity. With the quantitative CT measurements correlating with the severity of PE, it may be used to establish criteria for surgical treatment of PE patients or to evaluate lung parenchymal changes after surgery.

## Supporting information

S1 File (PDF)
