# Supplementary material for: Quantitative CT lung volumetry and densitometry in pediatric pectus excavatum
Source: PLoS One. 2024 Jul 23;19(7):e0299589. doi: 10.1371/journal.pone.0299589 (PMC11265689; doi:10.1371/journal.pone.0299589)
Supplement: S1 File — (PDF) [file pone.0299589.s001.pdf]

No	Dx	Ht(cm)	BW(kg)	Pectus I_insp	Pectus I_exp	Vol_insp	vol_exp
1	1	111.1	19.2	3.70	3.26	1059	791
2	1	111.5	21.0	3.64	3.95	1193	991
3	1	106.0	18.2	3.47	3.53	1073	1023
4	1	103.8	19.0	6.70	5.97	812	714
5	1	107.8	17.5	4.03	4.42	1428	974
6	1	114.0	18.0	5.50	4.55	1236	887
7	1	102.5	17.6	4.92	4.33	879	666
8	1	96.0	14.6	5.63	5.27	702	598
9	1	103.6	16.2	4.07	3.52	948	910
10	1	113.8	20.2	6.40	4.53	1580	960
11	1	98.0	17.0	4.15	4.65	776	761
12	1	105.0	18.7	3.95	4.56	880	741
13	1	106.0	17.9	3.97	4.62	1267	697
14	1	109.7	18.7	5.39	5.02	1031	918
15	1	104.0	16.6	4.92	5.17	971	750
16	1	77.0	8.4	3.37	3.46	1249	915
17	1	111.8	24.8	3.60	4.12	1020	899
18	1	125.0	26.0	4.08	3.98	1364	1160
19	1	106.4	19.2	4.23	4.20	1001	823
20	1	107.1	18.0	3.62	3.76	774	635
21	1	124.0	23.9	2.89	3.55	1939	1273
22	1	115.0	18.7	5.99	5.78	1139	1088
23	1	108.7	18.1	4.89	4.30	1136	848
24	1	105.7	17.2	4.71	4.88	1417	1249
25	1	110.1	18.9	3.81	3.67	1029	939
26	1	116.4	22.8	3.76	4.88	1182	793
27	1	117.0	22.0	4.02	4.31	1274	1119
28	1	111.0	20.0	4.68	5.72	1098	704
29	1	108.4	16.3	4.27	4.53	871	743
30	1	100.0	15.0	3.91	3.78	1151	923
31	1	106.0	16.0	4.52	4.21	1044	895
32	1	118.2	27.0	4.33	4.68	1792	1390
33	1	107.6	18.7	3.04	3.79	1463	972
34	1	117.0	29.9	3.90	3.73	1509	1355
35	1	116.3	20.2	5.27	4.56	1252	906
36	1	116.0	24.5	4.05	3.99	1008	983
37	1	112.1	16.4	4.02	4.31	886	835
38	1	110.0	16.8	3.46	4.49	1299	1001
39	1	109.7	17.3	3.65	4.56	1635	1155
40	1	110	18.5	2.88	3.08	1305	463
41	1	114	17.8	4.04	3.72	1529	1413
42	1	125.0	26.0	3.15	3.74	1489	984
43	1	117.4	20.6	3.31	3.76	1516	1058
44	1	109.7	17.8	3.37	3.31	1695	1610
45	1	126.7	26.9	4.75	4.94	1520	1226
46	1	119.8	23.5	4.07	4.75	1027	965
47	1	123.9	38.9	2.74	2.89	1339	1036
48	1	87.7	13.1	3.24	3.32	1447	1349
49	1	127.0	27.8	4.05	4.55	1721	1230
50	1	114.5	17.4	3.92	5.53	1876	1128
51	1	117.3	14.0	3.44	3.61	1741	1524
52	1	113	19.7	3.37	4.90	1581	707
53	1	117.8	18.1	4.13	3.84	1618	1308
54	1	124.2	22.4	2.97	3.04	1660	1513
55	1	123	25.6	5.71	5.34	1432	1052
56	1	125.0	23.0	4.66	5.72	1954	1451

57	1	129.9	29.7	4.56	4.18	1324	1151
58	1	131.9	41.5	3.20	3.71	1617	1066
59	1	124.9	24.4	3.90	3.40	1425	1285
60	1	122.1	26.8	4.27	4.54	1812	1172
61	1	126.6	24.5	3.52	3.74	1631	1459
62	1	118.7	21.1	3.92	4.85	1467	939
63	1	138.6	29.0	4.53	6.18	2441	1311
64	1	135.9	37.2	3.42	3.86	2255	1344
65	1	140.2	35.7	2.72	3.35	2701	2139
66	1	143.7	35.3	4.58	5.98	2069	1309
67	1	134.6	33.1	3.65	4.52	1650	1093
68	1	138.0	34.0	4.41	5.79	1711	1117
69	1	139.1	29.6	3.15	3.34	1938	1371
70	1	137.2	24.5	4.90	5.46	1762	1367
71	1	108	18.8	3.43	3.83	2320	1415
72	1	128	22.6	2.70	2.57	1741	1117
73	1	141.5	30.9	3.07	3.33	2459	1669
74	1	142.5	27.2	6.17	6.41	1916	1351
75	1	139.6	35	4.07	4.72	1948	1256
76	1	142	33	3.87	3.83	1631	1464
77	1	163.5	43.0	6.30	7.94	3182	2658
78	1	141.0	24.0	5.14	5.60	1558	1332
79	1	143.5	35.6	3.47	3.67	2257	1535
80	1	138.0	27.0	3.84	4.30	1867	1809
81	1	154.3	32.0	3.23	4.04	2972	1630
82	1	143	33	3.53	4.86	2625	1136
83	1	154.1	33.45	5.24	8.87	2291	1260
84	1	166.1	47.2	4.27	4.59	3744	2904
85	1	173.0	76.0	3.57	3.95	3440	2407
86	1	164.0	54.9	3.72	3.92	2509	2042
87	1	162.1	50.8	5.51	7.08	2794	1707
88	1	160.0	48.0	4.05	4.63	2979	2115
89	1	152.0	37.2	4.44	5.19	2765	1728
90	1	162.2	51.7	4.56	4.28	3993	3786
91	1	162	44	3.63	3.70	3614	3172
92	1	158.8	45.5	4.40	3.85	2604	1696
93	1	148.7	35.1	4.49	4.91	2270	1533
94	1	152.1	35.3	4.01	6.14	3256	1498
95	1	162.0	45.0	3.60	4.84	4167	2521
96	1	171.5	48.0	4.88	5.21	4018	2327
97	1	165.5	49.0	4.21	4.69	3189	2395
98	1	159.4	44.1	3.71	5.23	3684	2049
99	1	169.7	52.6	4.01	5.47	3838	1811
100	1	170.0	47.0	3.72	4.49	3750	2586
101	1	177.0	60.0	3.13	3.46	3696	2353
102	1	174.7	49.7	4.84	6.29	3378	2130
103	1	148.5	32.0	5.52	7.87	2430	1408
104	1	173.6	44.0	5.87	8.45	3916	2297
105	1	162.0	45.0	3.48	5.26	3717	1613
106	1	160.2	39.5	3.27	4.50	3305	1366
107	1	170.2	42.3	3.88	5.35	3711	2328
108	1	155.8	49.5	3.49	3.89	3087	1468
109	1	154.6	39.2	4.25	5.78	2478	1934
110	1	161.2	47	5.07	5.51	3238	3175
111	1	166	45	4.17	4.91	2723	1966
112	1	164.8	47.5	3.06	3.39	2831	1853
113	1	169.9	46.9	2.90	3.94	5727	3534
114	1	160.0	49.0	3.68	4.13	2720	1889
115	1	173.0	53.0	3.99	4.99	4282	1676

116	1	181.5	59.5	5.47	5.37	3881	3819
117	1	168.6	44.1	3.18	4.08	3912	2546
118	1	170.0	49.6	2.86	3.15	3097	2300
119	1	165.0	41.0	3.41	4.08	3736	1975
120	1	172.8	57.0	2.65	3.38	5263	2803
121	1	163.0	57.1	3.77	5.00	4203	2341
122	1	177.9	62.8	9.27	11.75	3257	2107
123	1	170.0	55.0	3.04	3.71	4302	2846
124	1	158.4	44.9	4.52	7.63	3333	1513
125	1	161.0	49.0	3.03	3.52	3536	2120
126	1	169.7	51.2	3.14	4.94	4529	2802
127	1	174.7	50.5	4.16	5.12	3646	2543
128	1	160.0	41.5	4.88	7.34	3573	1864
129	1	167.2	44.8	2.79	3.07	4169	3194
130	1	184.0	56.0	3.96	5.27	4617	3071
131	1	169.0	41.0	4.95	4.84	2619	2519
132	1	172.0	75.0	4.55	6.00	4161	2284
133	1	178.8	64.0	7.29	9.57	3833	2608
134	1	175.1	57.6	4.60	5.75	3817	2496
135	1	168.4	53.2	3.32	5.42	4686	2002
136	1	168.0	41.8	3.92	7.71	4074	1937
137	1	181.5	66.1	4.69	5.53	4815	3215
138	1	182.2	59.7	7.50	9.15	4073	3401
139	1	170.9	62.4	6.24	12.46	4109	1982
140	1	166.0	44.0	3.52	5.48	3820	2216
141	1	157.4	34	3.20	3.76	2736	1606
142	1	166.5	48.7	4.25	5.50	3196	1993
143	1	173.4	49.5	4.69	6.20	4036	2580
144	1	163.5	56.0	6.59	14.76	3914	2196
145	1	171.0	53.0	3.83	5.68	5178	2791
146	1	173.4	70.7	4.42	5.74	4671	2057
147	1	168.1	53.1	3.33	4.75	4686	1967
148	1	171.0	58.4	4.06	4.51	3757	2264
149	1	167.6	52.7	4.49	4.94	4211	3137
150	1	176.7	56.9	2.89	3.67	6034	3388
151	1	165	51	4.37	4.61	3201	2050
152	1	175.3	55.6	7.06	17.19	5499	2653
153	1	172.4	55.7	3.44	5.14	5818	2881
154	1	180.0	64.0	4.13	5.41	4968	2409
155	1	165.3	52.1	3.61	5.71	3939	1940
156	1	158.6	59.6	4.51	5.37	2973	1301
157	1	162	55	5.54	4.61	2687	1947
158	1	170.2	53.0	3.23	3.63	4742	3520
159	1	176.0	59.8	3.51	5.75	4862	2168
160	1	178.0	62.0	5.09	6.19	4370	3011
161	1	174.0	52.0	3.52	6.19	4666	2687
162	1	176.6	56.7	3.88	4.85	4160	2481
163	1	188.0	70.0	6.42	9.25	6314	4228
164	1	169.3	59.4	4.03	5.20	4085	2568
165	0	110	23.6			522	434
166	0	123	31.5			1822	808
167	0	119.0	27.3			2185	1141
168	0	127.2	20			1130	824
169	0	142.7	39			1716	1106
170	0	137	30			2664	2109
171	0	138	35.7			2063	914
172	0	141.2	30.5			1868	1536
173	0	137	39.9			2497	1279
174	0	137.0	38.0			2603	1493

175	0	138.4	32	2763	1146
176	0	162.4	47.7	3994	2772
177	0	165	75	2505	1858
178	0	162	52	3067	1503
179	0	154	57	2501	1408
180	0	162.5	49.0	3706	1849
181	0	159.0	44.0	3451	1484
182	0	149	40	2648	1058
183	0	167.2	65	3897	1934
184	0	169.8	82.7	1585	1088
185	0	174	55.3	4889	3074
186	0	160.6	37.9	4278	2585
187	0	153	51	2783	1535
188	0	174	85	2792	1342
189	0	175.9	46.0	4052	2812
190	0	160	60	3434	1574
191	0	169	61	5395	1957
192	0	173	54.4	3383	1980
193	0	180.0	60.0	4286	3210
194	0	163.6	68.6	5121	2282
195	0	166	65	3803	1916
196	0	161.4	50	4033	1262
197	0	175.4	89.5	5707	3509
198	0	166	57	3151	1934
199	0	193.3	101.0	6391	2380

density_insp	density_exp	vol diff (I-E)	density dif (I-E)	vol ratio (E/I)	density ratio (E/I)
686	600	268	86	0.75	0.87
699	653	202	46	0.83	0.93
770	763	50	7	0.95	0.99
632	613	98	19	0.88	0.97
768	675	454	93	0.68	0.88
776	696	349	80	0.72	0.90
695	603	213	92	0.76	0.87
667	624	104	43	0.85	0.94
694	679	38	15	0.96	0.98
782	679	620	103	0.61	0.87
637	626	15	11	0.98	0.98
717	662	139	55	0.84	0.92
762	589	570	173	0.55	0.77
712	685	113	27	0.89	0.96
707	643	221	64	0.77	0.91
742	657	334	85	0.73	0.89
737	710	121	27	0.88	0.96
712	663	204	49	0.85	0.93
719	675	178	44	0.82	0.94
650	605	139	45	0.82	0.93
789	690	666	99	0.66	0.87
732	724	51	8	0.96	0.99
743	675	288	68	0.75	0.91
816	790	168	26	0.88	0.97
702	675	90	27	0.91	0.96
728	613	389	115	0.67	0.84
711	674	155	37	0.88	0.95
711	594	394	117	0.64	0.84
668	637	128	31	0.85	0.95
729	675	228	54	0.80	0.93
769	725	149	44	0.86	0.94
805	769	402	36	0.78	0.96
777	680	491	97	0.66	0.88
778	754	154	24	0.90	0.97
729	644	346	85	0.72	0.88
640	630	25	10	0.98	0.98
656	646	51	10	0.94	0.98
777	720	298	57	0.77	0.93
817	721	480	96	0.71	0.88
757	451	842	306	0.35	0.60
825	809	116	16	0.92	0.98
778	687	505	91	0.66	0.88
768	674	458	94	0.70	0.88
820	812	85	8	0.95	0.99
750	711	294	39	0.81	0.95
672	662	62	10	0.94	0.99
737	655	303	82	0.77	0.89
755	748	98	7	0.93	0.99
776	692	491	84	0.71	0.89
827	730	748	97	0.60	0.88
781	756	217	25	0.88	0.97
818	617	874	201	0.45	0.75
797	767	310	30	0.81	0.96
765	744	147	21	0.91	0.97
756	677	380	79	0.73	0.90
803	742	503	61	0.74	0.92

722	679	173	43	0.87	0.94
756	660	551	96	0.66	0.87
708	687	140	21	0.90	0.97
789	716	640	73	0.65	0.91
785	764	172	21	0.89	0.97
791	690	528	101	0.64	0.87
818	684	1130	134	0.54	0.84
797	675	911	122	0.60	0.85
844	802	562	42	0.79	0.95
792	699	760	93	0.63	0.88
739	622	557	117	0.66	0.84
767	662	594	105	0.65	0.86
786	711	567	75	0.71	0.90
755	704	395	51	0.78	0.93
827	751	905	76	0.61	0.91
797	706	624	91	0.64	0.89
806	727	790	79	0.68	0.90
766	682	565	84	0.71	0.89
804	716	692	88	0.64	0.89
753	728	167	25	0.90	0.97
796	758	524	38	0.84	0.95
740	715	226	25	0.85	0.97
785	708	722	77	0.68	0.90
806	800	58	6	0.97	0.99
824	691	1342	133	0.55	0.84
815	644	1489	171	0.43	0.79
813	659	1031	154	0.55	0.81
827	785	840	42	0.78	0.95
825	761	1033	64	0.70	0.92
737	701	467	36	0.81	0.95
786	676	1087	110	0.61	0.86
779	701	864	78	0.71	0.90
782	679	1037	103	0.62	0.87
840	820	207	20	0.95	0.98
835	825	442	10	0.88	0.99
780	703	908	77	0.65	0.90
790	699	737	91	0.68	0.88
833	669	1758	164	0.46	0.80
834	733	1646	101	0.60	0.88
839	741	1691	98	0.58	0.88
791	738	794	53	0.75	0.93
847	734	1635	113	0.56	0.87
823	651	2027	172	0.47	0.79
823	756	1164	67	0.69	0.92
791	690	1343	101	0.64	0.87
796	684	1248	112	0.63	0.86
795	683	1022	112	0.58	0.86
832	744	1619	88	0.59	0.89
838	663	2104	175	0.43	0.79
838	633	1939	205	0.41	0.76
840	761	1383	79	0.63	0.91
839	699	1619	140	0.48	0.83
805	741	544	64	0.78	0.92
823	814	63	9	0.98	0.99
766	695	757	71	0.72	0.91
801	697	978	104	0.65	0.87
880	813	2193	67	0.62	0.92
797	723	831	74	0.69	0.91
815	596	2606	219	0.39	0.73

760	757	62	3	0.98	1.00
831	753	1366	78	0.65	0.91
795	732	797	63	0.74	0.92
848	742	1761	106	0.53	0.88
858	747	2460	111	0.53	0.87
840	736	1862	104	0.56	0.88
753	661	1150	92	0.65	0.88
814	730	1456	84	0.66	0.90
798	634	1820	164	0.45	0.79
805	686	1416	119	0.60	0.85
842	754	1727	88	0.62	0.90
806	735	1103	71	0.70	0.91
835	709	1709	126	0.52	0.85
842	806	975	36	0.77	0.96
850	779	1546	71	0.67	0.92
765	762	100	3	0.96	1.00
823	717	1877	106	0.55	0.87
805	705	1225	100	0.68	0.88
825	738	1321	87	0.65	0.89
868	694	2684	174	0.43	0.80
848	705	2137	143	0.48	0.83
816	749	1600	67	0.67	0.92
799	767	672	32	0.84	0.96
789	646	2127	143	0.48	0.82
855	748	1604	107	0.58	0.87
833	739	1130	94	0.59	0.89
817	735	1203	82	0.62	0.90
834	759	1456	75	0.64	0.91
825	706	1718	119	0.56	0.86
861	759	2387	102	0.54	0.88
806	647	2614	159	0.44	0.80
862	677	2719	185	0.42	0.79
798	700	1493	98	0.60	0.88
832	782	1074	50	0.74	0.94
842	730	2646	112	0.56	0.87
800	708	1151	92	0.64	0.89
850	709	2846	141	0.48	0.83
870	748	2937	122	0.50	0.86
822	666	2559	156	0.48	0.81
852	686	1999	166	0.49	0.81
808	625	1672	183	0.44	0.77
794	727	740	67	0.72	0.92
812	762	1222	50	0.74	0.94
849	679	2694	170	0.45	0.80
809	738	1359	71	0.69	0.91
854	755	1979	99	0.58	0.88
795	677	1679	118	0.60	0.85
849	780	2086	69	0.67	0.92
824	736	1517	88	0.63	0.89
575	534	88.00	41.00	0.83	0.93
838	656	1014.00	182.00	0.44	0.78
823	687	1044.00	136.00	0.52	0.83
684	590	306.00	94.00	0.73	0.86
736	630	610.00	106.00	0.64	0.86
851	813	555.00	38.00	0.79	0.96
830	660	1149.00	170.00	0.44	0.80
767	715	332.00	52.00	0.82	0.93
823	676	1218.00	147.00	0.51	0.82
847	751	1110.00	96.00	0.57	0.89

834	654	1617.00	180.00	0.41	0.78
844	777	1222.00	67.00	0.69	0.92
737	630	647.00	107.00	0.74	0.85
805	635	1564.00	170.00	0.49	0.79
782	646	1093.00	136.00	0.56	0.83
810	649	1857.00	161.00	0.50	0.80
828	617	1967.00	211.00	0.43	0.75
851	669	1590.00	182.00	0.40	0.79
829	675	1963.00	154.00	0.50	0.81
648	579	497.00	69.00	0.69	0.89
828	748	1815.00	80.00	0.63	0.90
859	780	1693.00	79.00	0.60	0.91
809	669	1248.00	140.00	0.55	0.83
772	593	1450.00	179.00	0.48	0.77
824	763	1240.00	61.00	0.69	0.93
834	675	1860.00	159.00	0.46	0.81
868	662	3438.00	206.00	0.36	0.76
825	713	1403.00	112.00	0.59	0.86
826	770	1076.00	56.00	0.75	0.93
844	704	2839.00	140.00	0.45	0.83
772	628	1887.00	144.00	0.50	0.81
858	606	2771.00	252.00	0.31	0.71
848	761	2198.00	87.00	0.61	0.90
804	683	1217.00	121.00	0.61	0.85
860	656	4011.00	204.00	0.37	0.76
